# Dual Seasonal Patterns for Influenza, China

**DOI:** 10.3201/eid1604.091578

**Published:** 2010-04

**Authors:** Yue-Long Shu, Li-Qun Fang, Sake J. de Vlas, Yan Gao, Jan Hendrik Richardus, Wu-Chun Cao

**Affiliations:** Chinese Center for Disease Control and Prevention, Beijing, People’s Republic of China (Y.L. Shu, Y. Gao); Beijing Institute of Microbiology and Epidemiology, Beijing (L.-Q. Fang); University Medical Center Rotterdam, Rotterdam, the Netherlands (S.J. de Vlas, J.H. Richardus); State Key Laboratory of Pathogen and Biosecurity, Beijing (W.-C. Cao); 1 These authors contributed equally to this article.

**Keywords:** Influenza, seasonal pattern, surveillance, viruses, letter

**To the Editor:** Since 2000, the People’s Republic of China has had a nationwide surveillance network for influenza, which as of 2005 has been reported on the Chinese Center for Disease Control and Prevention website (www.cnic.org.cn/ch/). This surveillance has shown a remarkable dual pattern of seasonal influenza on mainland China. Whereas a regular winter pattern is noted for northern China (similar to that in most parts of the Northern Hemisphere), the pattern in southern China differs. In southern China, influenza is prevalent throughout the year; it has a clear peak in the summer and a less pronounced peak in the winter. Because this dual seasonal pattern of influenza has not been reported outside China and is relevant to pandemic (H1N1) 2009, we describe surveillance data for rates of consultation for influenza-like illness (ILI) and influenza subtypes in patients with ILI. We emphasize the spread of influenza from southern to northern China.

Before it was extended in June 2009, the National Influenza Surveillance Network had been composed of 63 influenza laboratories and 197 sentinel hospitals across 31 provinces of mainland China. In 13 of 16 northern provinces, surveillance began from the week including October 1 and ended in the week including March 31 of the following year. In the 3 northern provincial areas of Liaoning, Tianjin, and Gansu and in all southern provinces, surveillance was conducted throughout the year. Data consisted of information about ILI cases and virus subtypes. The sentinel hospitals defined ILI cases according to World Health Organization criteria: sudden onset of fever >38°C, cough or sore throat, and absence of other diagnoses (*1*). The number of ILI cases and the total number of outpatients at the sites (ILI consultation rate) were recorded each day and reported to the National Influenza Surveillance Information System each week.

Sentinel hospitals were required to collect 5–15 nasopharyngeal swabs each week from ILI patients who had not taken antiviral drugs and who had fever (>38°C) for no longer than 3 days. The swabs were sent to the corresponding influenza laboratories for virus isolation and identification; results were reported to the National Influenza Surveillance Information System within 24 hours.

From the National Influenza Surveillance Network, a database of surveillance information from April 2006 to March 2009 was established. For influenza surveillance purposes, mainland China was divided into northern and southern parts, basically following the Qinling Mountain range in the west and the Huai River in the east. The prominent influenza peaks in the winter in the north and summer in the south were clear for adults and for children (Figure, panel A); the level of ILI was 3–5× for children. The influenza subtypes causing the 3 peaks in the north were preceded by a peak of the same subtypes in the south. During winter 2006–07, the influenza subtype was seasonal H1N1 and to a lesser extent H3N2. In winter 2007–08, the virus was B/Yamagata; and in 2008–09, it was again seasonal influenza A (H1N1), which was almost absent in the south during April–December 2007. Antigenic characteristics of the influenza virus from the north were similar to those from the south in the same epidemic episode (*2*). Furthermore, influenza A (H3N2) was in southern China throughout the year, whereas in northern China, this subtype only showed a clear peak in the first 2 winters of the study period. Subtype B/Victoria and B (unsubtyped) were both in northern and southern China in irregular and low numbers. Data from the 3 northern provincial areas with year-round surveillance confirmed that influenza cases during April–September were negligible (data not shown).

**Figure:**
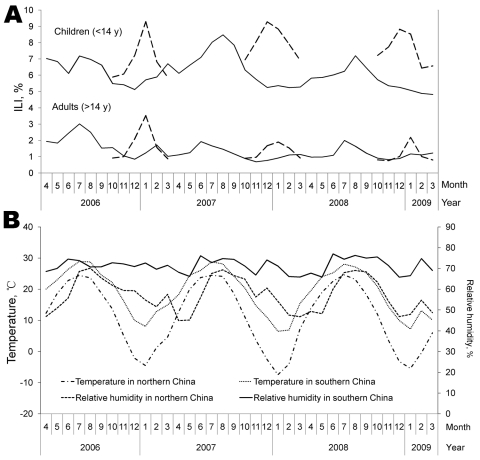
Epidemic patterns, by month, from the surveillance data of the influenza-like illness (ILI) consulting rate and influenza subtypes in ILI patients in mainland China, per month, April 2006–March 2009. A) ILI percentages for northern (dashed lines) and southern (solid lines) mainland China, by age group. B) Average temperature and relative humidity.

The influenza subtypes of seasonal influenza A (H1N1) and B/Yamagata that have caused the past 3 summer peaks in southern China were followed by an epidemic of the same subtypes in northern China during the subsequent winter. This finding may indicate that these peaks are regular epidemic phenomena for seasonal influenza in China. Another possible explanation is that other subtypes were cocirculating with the predominant subtype at the time of epidemics.

The dual pattern of seasonal peaks for influenza is well-known for the Northern and Southern Hemispheres, but apparently it is also possible on 1 side of the equator. China is a large country with climatic differences between north and south. Although most of southern China is above the Tropic of Cancer, it is warmer and more humid than northern China (Figure, panel B), which may explain the different seasonal patterns within mainland China (*3*). Knowledge of the dual patterns of influenza in China is relevant for determining effective control measures, and knowledge of the underlying mechanisms of such patterns is relevant to understanding the epidemiology of influenza in general.

## References

[1] World Health Organization. WHO recommended surveillance standards, Second edition [cited 2009 Aug 19]. http://www.who.int/csr/resources/publications/surveillance/WHO_CDS_CSR_ISR_99_2_EN/en/

[2] Zhang Y, Antigenic and genetic study of influenza virus circulated in China in 2006 [in Chinese] [Medline]. Zhonghua Shi Yan He Lin Chuang Bing Du Xue Za Zhi. 2007;21:304–6. 18322584

[3] Chan PK, Mok HY, Lee TC, Chu IM, Lam WY, Sung JJ. Seasonal influenza activity in Hong Kong and its association with meteorological variations. J Med Virol. 2009;81:1797–806. 10.1002/jmv.21551 19697414

